# Different Lengths of Percutaneous Transverse Iliosacral Screw in Geometric Osseous Fixation Pathway: A Finite-Element Analysis

**DOI:** 10.1007/s43465-022-00656-x

**Published:** 2022-06-01

**Authors:** Qiong Wu, Yuanzhi Zhang, Shaobai Wang, Rui Liu, Gang Liu

**Affiliations:** 1 Department of Radiology, The Affiliated Hospital of Inner Mongolia Medical University, Hohhot, People’s Republic of China; 2 Department of Orthopaedics, The Affiliated Hospital of Inner Mongolia Medical University, Hohhot, People’s Republic of China; 3 School of Kinesiology, Shanghai University of Sport, Shanghai, People’s Republic of China

**Keywords:** Osseous fixation pathways, Internal fixation, Finite element analysis

## Abstract

**Objective:**

To evaluate the biomechanical performances of the sacroiliac screw fixation of the first sacral vertebra with different lengths of screws using the Finite-Element Method.

**Methods:**

First, pelvic CT images were generated from a healthy volunteer, and multislice sagittal views were produced to determine the axis for the first sacral vertebra geometric osseous fixation pathway (GOFP). Subsequently, according to the geometric size and mechanical parameters of the iliosacral screw, the screw models with the same diameter of 7.3 mm and different lengths of 80 mm, 90 mm, 100 mm, 110 mm, 120 mm, 130 mm and 140 mm were built. Then the seven screws were assembled with the pelvic model. The maximum von Mises stress and the shape variables were evaluated for the pelvis and the screws.

**Results:**

Results are shown for the pelvic and GOFP screw, respectively. The simulation results show that the maximum von Mises stress in the cortex of the pelvic ring of the pelvis with the 130-mm length screw is the lowest among the pelvic models with different screws. Moreover, the peak displacement of the pelvis with the 130-mm length screw is the smallest. These results indicate that under the standing condition, a 130-mm length screw can decrease the stress concentration and result in a more effective transfer of stress within the reconstructed pelvis. In addition, the displacement of the screw with a 130-mm length is the smallest among all the considered screws. The peak von Mises stresses in the 130-mm length screw and the cortex are still within a low and elastic range below the yielding strengths of the materials.

**Conclusion:**

Through the finite element analysis, the GOFP can be used as a safe and effective way for iliosacral screw fixation. The optimal length of the screw may be 130 mm length.

## Introduction

Percutaneous iliosacral (IS) screw fixation is a common surgical intervention method for longitudinal sacral fractures that is used to stabilize pelvic and acetabular fractures. However, due to the complicated structure in the pelvis and the deep location, this treatment requires high accuracy in screw implantation [1–3]. S1 screw fixations are most commonly used during surgery, by two means of fixation. First is oblique fixed, i.e., along the direction of S1 pedicle; second, strictly in accordance with the S1 fixed inlet and outlet diameter, which takes place in a small space. This requires substantial experience and detailed anatomical knowledge to find the proper entry point and trajectory even with the use of a navigation system [4]. Gardner’s study found that 4 degree deviation in iliosacral screw placement can result in entering of S1 vertebral foramen, or piercing the front cortex of the sacrum. The piercing screws can cause potentially vessels and sacral nerve injury, up to 2–15% of vascular, lumbosacral trunk, and cauda equina injury during surgery. The iliosacral overall screw placement position error rate was 2.05–13% [5–7]. Bishop and Routt were the first authors to conceptualize the opportunities for fixation about the pelvis and acetabulum as pelvic osseous fixation pathways (OFPs) [8]. These geometrically complex “bone tubes” are simply corticated bony cylinders of different dimensions and orientations that can accommodate intraosseous implants. Commonly, screws are used to fill the available OFP and thereby stabilize pelvic and acetabular fractures either percutaneously or after a formal open approach. The screw numbers, locations, diameters, and lengths selected are dependent on the individual patient’s osteology, assuming that an accurate fracture reduction has been achieved. Recently, there have been numerous studies investigating the method of fixing percutaneous IS screws in the first sacral segment (S1) for sacral fractures. However, most of these methods require the assistance of an intra-operative navigation system. Without these instruments, it is difficult to fix the percutaneous IS screw freehand under fluoroscopy given the lack of a direct view and limited tactile control. These obstacles also impact the precision of any intervention. Moreover, anatomical variations of the pelvis increase the risk of misinterpretation and error [9–11]. The situation inevitably exposes surgeons and patients to excessive levels of ionizing radiation [12, 13]. If the optimal OFP for the percutaneous IS screw could be planned and defined prior to surgery, the operation would be both simplified and safer.

In this study, we demonstrated a computer-aided method for determining an optimal OFP for percutaneous IS screw fixation. The biomechanical performances of the sacroiliac screw fixation of the first sacral vertebra with different lengths of screws were evaluated using the Finite-Element Method (FEM).

### Ethics Statement

Ethical approval was obtained from the Human Research Ethics Committee, The Affiliated Hospital of Inner Mongolia Medical University, Hohhot, China.

## Materials and Methods

**Fig. 1:**
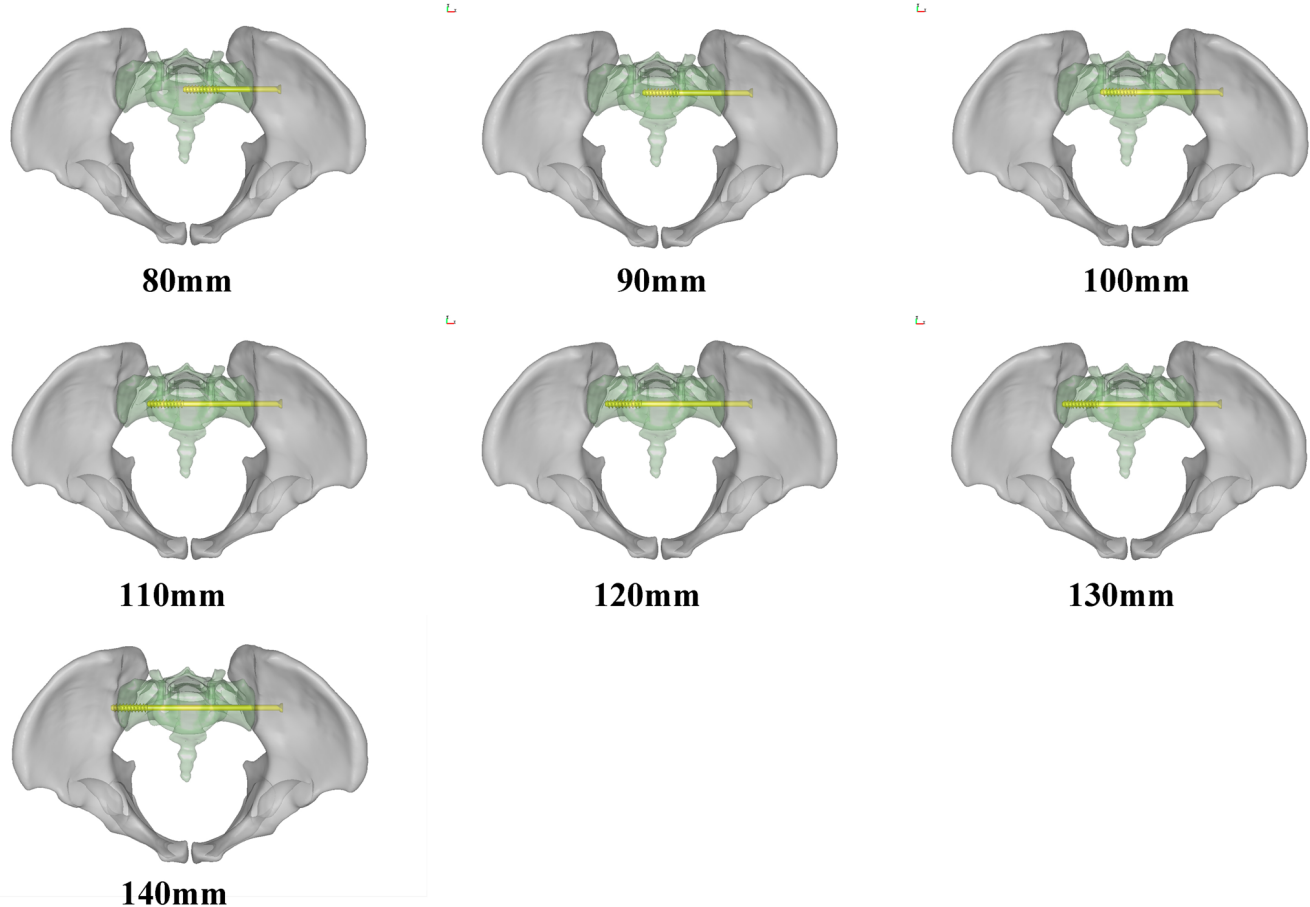
Assembly of the pelvic and different lengths of iliosacral screw model

A healthy male adult volunteer (age: 30 years, height: 175 cm, weight: 70 kg) was enrolled in this study. No tumors and severe bony deformities were diagnosed prior to the study. The pelvis was scanned using a spiral CT scanner (Light Speed 64; GE, Boston, MA, USA) at 120 kV with a slice thickness of 0.625 mm and a matrix of 512 × 512 pixels. The generated images were converted into DICOM format and were further processed using medical imaging software (Mimics Innovation Suite 15.0; Materialise, Leuven, Belgium) to obtain the STL format files for the 3D reconstruction of the pelvises. Visualization software (Imageware 12.0; EDS, Plano, TX, USA) was subsequently used to produce multi-slice sagittal views (thickness of 1.0 mm) of the 3D reconstruction images. First, we defined the geometric boundary of the safe zone on each sagittal view for the first sacral vertebra. Thus, the inscribed ellipse of the boundary was obtained from each view. Subsequently, the X, Y, and Z coordinates of the center in each inscribed ellipse (i.e., the intersection of the major and minor axes) were calculated. Finally, the least-squares methods [14] were used to fit the optimal axis that pass through the centers of the inscribed ellipses (a statistical procedure to find the best fit for a set of data points by minimizing the sum of the offsets or residuals of points from the line). This axis was defined as the optimal and safe pathway for the IS screw (i.e., the geometric osseous fixation pathway, GOFP). According to the geometric size and mechanical parameters of the iliosacral screw, seven screw models with the same diameter of 7.3 mm and different lengths of 80 mm, 90 mm, 100 mm, 110 mm, 120 mm, 130 mm, and 140 mm were built using the Solidworks 2012 software (Dassauh Systemes; France). Screws models were, respectively, assembled with the pelvic model according to the determined GOFP, which are illustrated in Fig. 1.

To perform the simulations, we defined the properties of the materials [15], such as the Elastic Modulus, and Poisson’s ratio of all parts of the digital models (Table 1). The entire pelvis FEA model was node-based smoothed finite-element method [16]. In total, the pelvic model contained 735,512 elements and 270,363 nodes. Cortical bone, cancellous bone, and internal fixation materials are solid32 units.

**Table 1:** Material properties

Component	Material	Young’s modulus	Pine ratio
Cortical bone	Cortical bone	1.5E + 10	0.3
Cancellous	Cancellous	5.0E + 8	0.2
Bone screw	Ti_6_Al_4_V	1.10E + 11	0.3

After performing the mesh control of each part, to ensure the perfect contact between the different structures, application regions of the loads onto the Z, X, and Y axes were set along of sagittal, coronal, and axial planes, respectively [17]. A 500 N load was applied to the region in the Z-axis direction [15, 18], and no loading was applied onto the X and Y axes. The freedom degrees on the articular surface of bilateral sacroiliac joints are constrained. The FEM analysis was performed in the ANSYS12.0 software (ANSYS, USA), the maximum von Mises stress and shape variables of the pelvis and the screws were evaluated. The mesh convergence was performed on a simplified FE model of a V-threaded screw with surrounding cancellous and cortical bone [19]. The screw–bone interface was modeled by a hard contact pair using surface-to-surface contact elements in combination with the penalty algorithm with a normal contact stiffness of 500 N/mm and a friction coefficient of zero.

## Results

**Fig. 2:**
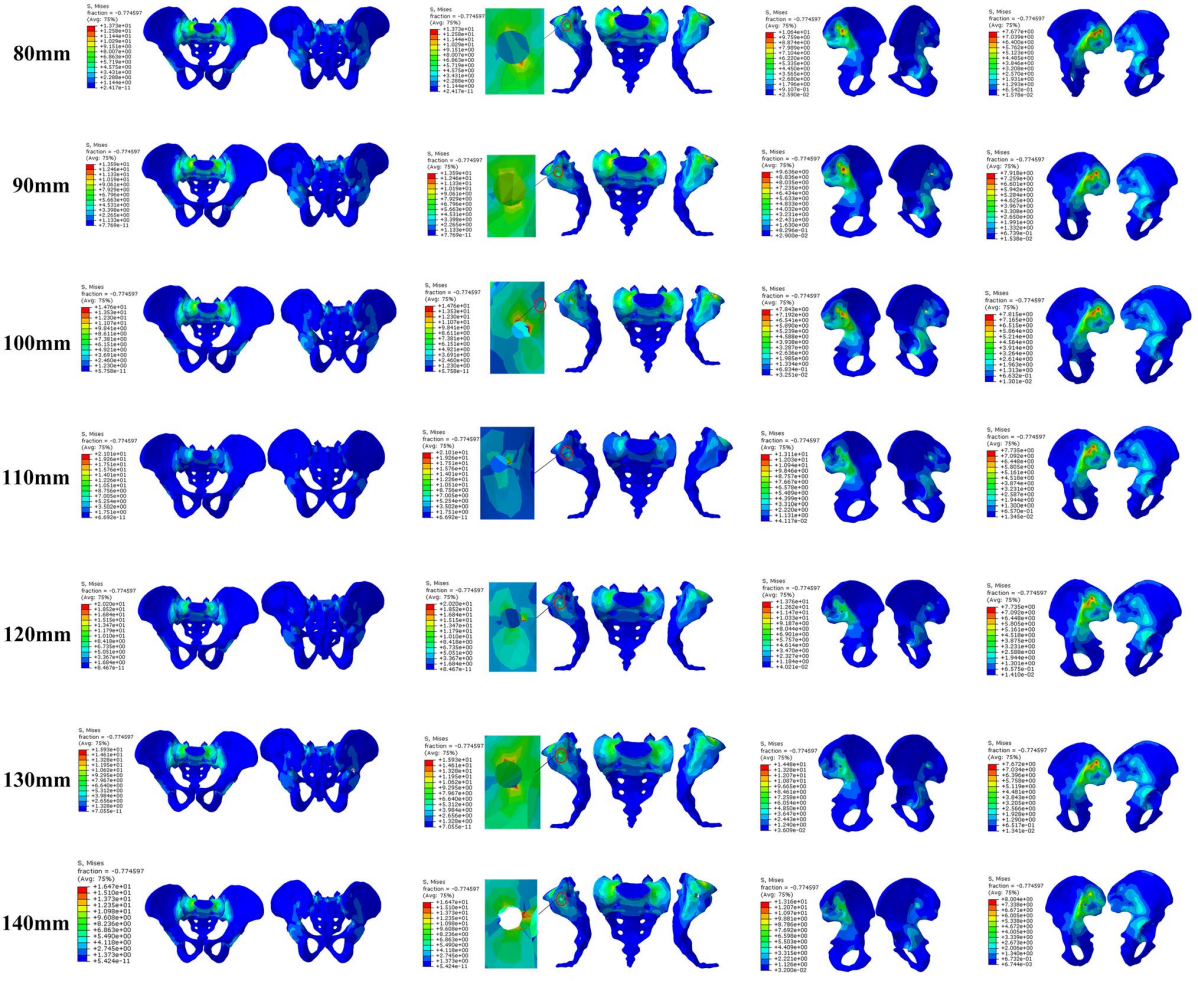
The Von Mises stress distribution on pelvic with different lengths of iliosacral screw fixation

**Fig. 3:**
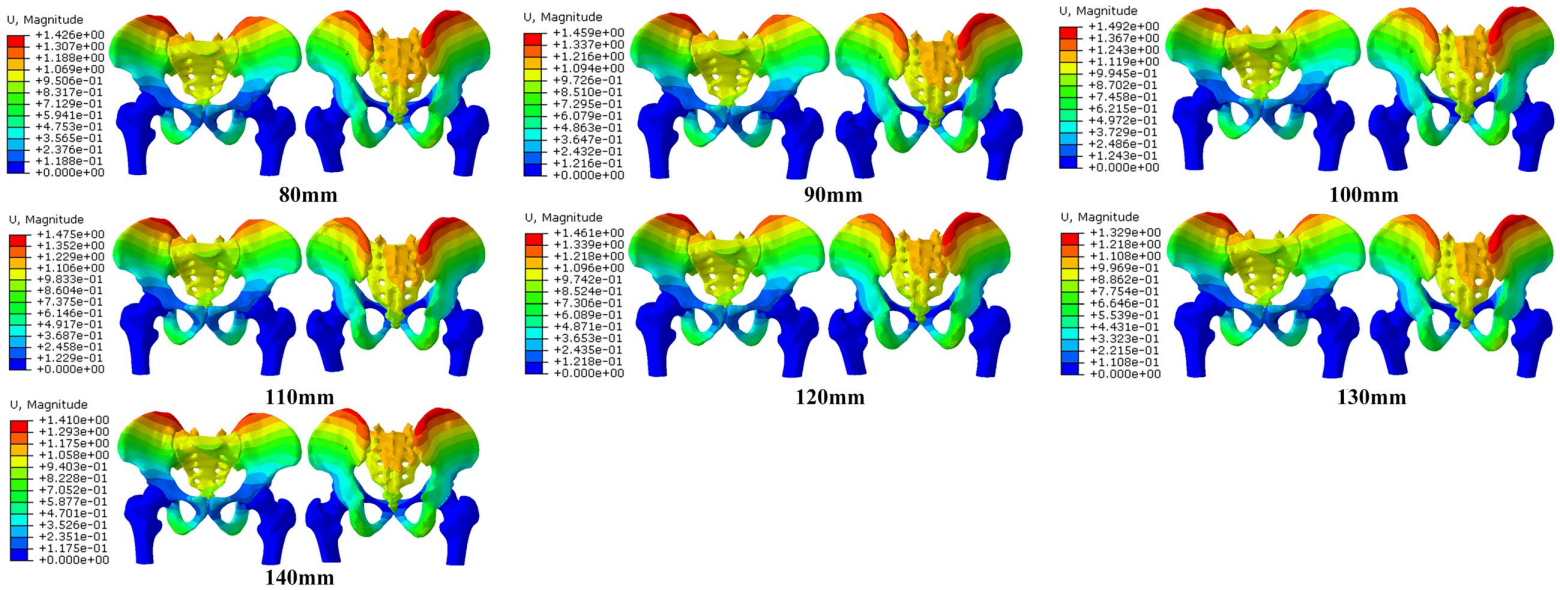
The displacement of pelvic with different lengths of iliosacral screw fixation

**Fig. 4:**
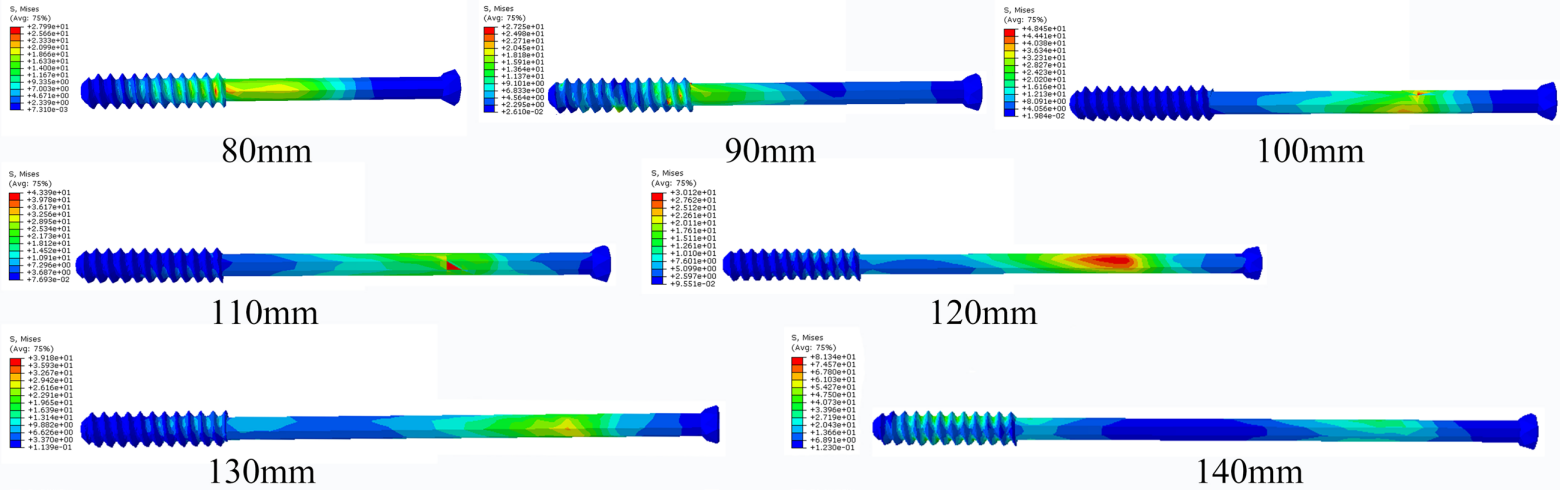
The Von Mises stress distribution on different lengths of iliosacral screw

**Fig. 5:**
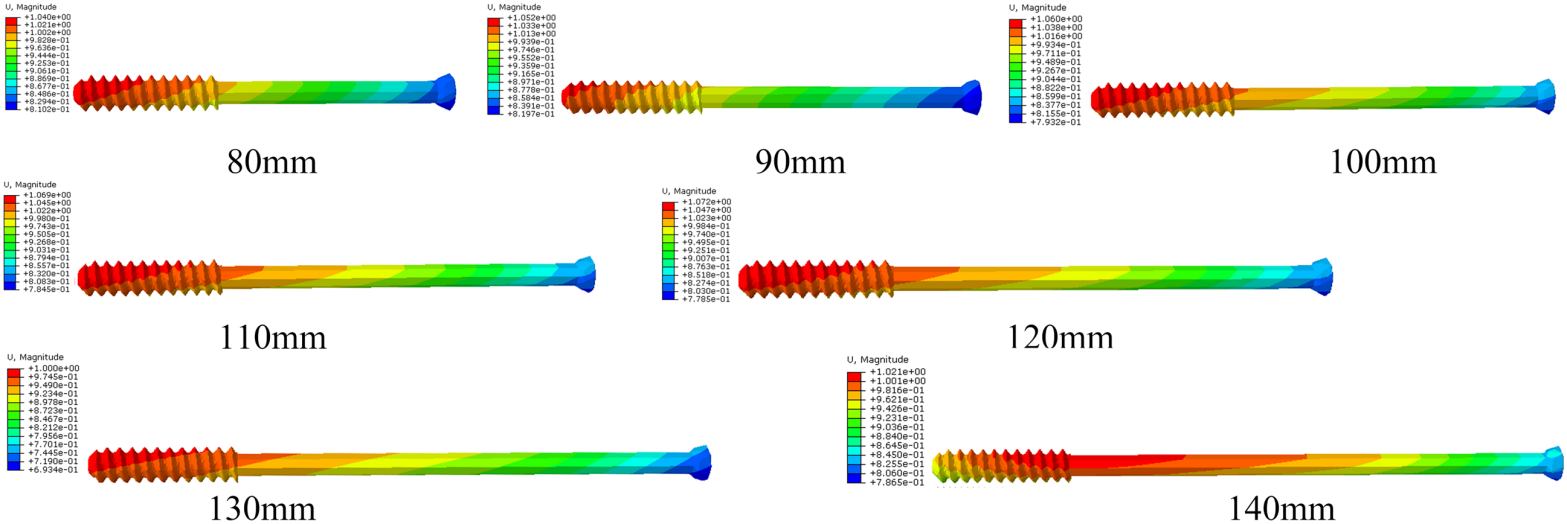
Displacement of different lengths of iliosacral screw

Among them, the screw displacement of 120 mm length was the largest, and the screw displacement of 130-mm length was the smallest, while the displacement increased when the length of the screw increased to 140 mm. The von Mises stress distribution in the pelvis showed that the maximum stress in the cortex of the pelvic ring with the screw length of 110 mm was the highest among all pelvic models. When the screw length was 130 mm, the stress decreased to a low level, and the stress increased when the screw length is 140 mm., The results indicate that the cortex of the pelvis with the screws of 110 mm and 120 mm length was prone to stress concentration and fracture, while the 130-mm length screw can reduce the stress concentration in the cortex and result in effective transferring of stress within the pelvis (Figs. 2, 3, 4, 5; Tables 2, 3, 4, 5).

**Table 2:** Von Mises stress in the cortex of the pelvis

Pelvis type	80 mm	90 mm	100 mm	110 mm	120 mm	130 mm	140 mm
Peak stress (MPa)	13.73	13.59	14.76	21.01	20.20	15.93	16.47
Position of peak stress	On the left sacroiliac articular surface of sacrum, inferior rim of the screw hole	On the left sacroiliac articular surface of sacrum, inferior rim of the screw hole	On the left sacroiliac articular surface of sacrum, posterior rim of the screw hole	On the left sacroiliac articular surface of sacrum, posterior rim of the screw hole	On the left sacroiliac articular surface of sacrum, posterior rim of the screw hole	On the left sacroiliac articular surface of sacrum, superior and inferior rim of the screw hole	On the left sacroiliac articular surface of sacrum, posterior rim of the screw hole

**Table 3:** Von Mises stress in the screws

Screw type	80 mm	90 mm	100 mm	110 mm	120 mm	130 mm	140 mm
Peak stress (MPa)	27.99	27.25	40.45	43.39	30.12	39.18	81.34
Position of peak stress	Inferior edge of the first thread	Inferior edge of the second thread	1/3 The length from the tap	1/3 The length from the tap	1/3 The length from the tap	1/4 The length from the tap	Inferior edge of the forth thread

**Table 4:** Displacement of the pelvis

Pelvis type	80 mm	90 mm	100 mm	110 mm	120 mm	130 mm	140 mm
Displacement (mm)	1.426	1.459	1.492	1.475	1.461	1.329	1.410

**Table 5:** Displacement of the screws

Screw type	80 mm	90 mm	100 mm	110 mm	120 mm	130 mm	140 mm
Displacement (mm)	1.040	1.052	1.060	1.069	1.072	1.000	1.021

## Discussion

For accurate IS screw placement, more studies were done on the structure and boundary of bony in the safety area, especially the analysis of projection or image based on CT [11, 20, 21]. Mendel et al. [17] reconstructed 3D pelvic CT of 85 adult patients. They extracted the triangle projection sacral S1 lateral edge, sacral promontory, and the leading edge of the iliosacral joint is mapped via the sacral auricular surface maximum set nail “safe passage”. The conclusion was the lateral sacral triangle in the lateral view which represents a simple and accurate preoperative method of support for the surgeon’s decision to undertake this procedure. No additional technical effort is necessary. Furthermore, Mendel and Radetzki [22] carried out to confirm the three-dimensional visualization of CT data and estimates based on bilateral IS screw placement average volume of space and screws into the bone pathway distribution points in the iliac surface area, to determine the three-dimensional image pathway optimization. The secure corridor for the IS screw is double cone-shaped, which has previously been described as a ‘‘vestibule’’ or ‘‘bottleneck’’. The optimal screw pathway with the greatest safety distance was 14.2 mm; the mean corridor length was 14.9 cm. However, these studies stayed in anatomical measurement, yet did not apply the results to the biomechanical analysis.

Cruz’s research shows that there was a significant size difference in the analyzed sacral vertebra, differing on a wider size in men than in women. However, there was no significant statistical difference between vertebral size and age. Age does not influence the width of the surgical corridor. The surgeon has a safe corridor considered narrower when inserting screws in a female pelvis than when in a male one [23].

To ensure the safety of the premise of the iliac body, it is recommended to use a long-threaded screw. Sagi [24] compared the sacrum and iliac wing body extraction forces of three threaded cancellous bone screws with various lengths after iliosacral screw fixation. They found that the long-threaded screw in the sacral body requires ten times extraction forces than the short thread in the sacral wing. The S1 pedicle screw fixation of the iliosacral joint must choose different screw length and path considering the variation in patient species, age, sex, and other factors.

The screw path was defined as McLaren’s methods [17]. The path extended from the outer table of one posterior ilium, across the near SI joint, through the S1 sacral segment, across the contralateral SI joint, and out the outer table of the opposite ilium. The diameter of the path was increased until it contacted and breached the thickness of the cortex in at least three locations. The results of our study show that under a given loading force of pelvic by stress and pelvic deformation increase gradually. The screw displacement of 120 mm length was the largest, and the screw displacement of 130-mm length was the smallest. The displacement increased again with the screw length of 140 mm. When the screw length was 130-mm length, the stress in the cortex of the pelvic ring decreased to a low level. Compared with Chen’s results [25], it is similar, so this finite-element model is effective.

Whether the optimal anatomical pathway obtained by our algorithm is also the optimal biomechanical solution is still not fully understood and should be verified in the subsequent studies. Furthermore, the optimal pathway provides a maximum safe range, rather than the surgery screw pathways. As long as the screw stayed within the pathways, its safety and reliability should be guaranteed.

Fradet’s study had shown that sacroiliac screws provided forces to failure 2.75 times higher than sacral fixation screws [26]. On the contrary, the initial stiffness was approximately half as much for sacroiliac screws. High stresses were located at screw tips for the sacral trajectories and near the cortical bone screw entry points for the sacroiliac trajectory. Overall, the diameter and length of the screws had significant effects on the screw fixation. However, this experiment was not carried out in our study. Although some scholars believe that sacroiliac joint stress and angular motion increases as ligament stiffness decreases. Periarticular intraligamentous strains vary depending on the magnitude and direction of the applied loads. Maximum ligamentous strains occur at the interosseous sacroiliac ligament [27], but Lee’s tests showed that the maximum pelvic stress in the finite-element models with muscles and ligaments was similar to that in the finite-element models without muscles and ligaments [28]. This pelvis without the muscles and ligaments has been proven to have similar mechanical properties to the human pelvis by other researchers [29, 30]. Therefore, the muscles and ligaments were not considered in this study, that is our limitation.

The lower bone density (vs. normal bone density) led to a decrease of the IS (20–35%) and force to failure (5–20%) values, which is consistent with published experimental results showing decrease of IS by 40% and force to failure by 30% for a 30% density reduction [31, 32]. By extending the control model to include the pelvis using a similar computational approach, the biomechanical simulation results are thus assumed to be generally relevant.

## Limitations

Some limitations should be considered when evaluating the simulation results. In this study, a pelvic model based on a healthy volunteer was used to build the finite-element model for evaluating the mechanical performance of iliosacral screws in the fixation of the first sacral vertebra. The effect of osteoporosis on the evaluation will be considered in future work. Also, to make the simulation results more convincing, the grid convergence analysis, and axial pullout and flexion/extension toggle forces on the screws representing intra and post-operative loads for complete biomechanical analysis should be calculated, these studies will be carried out in the next work.

## References

[1] Sathy AK, Starr AJ, Smith WR, Elliott A, Agudelo J, Reinert CM, Minei JP (2009). The effect of pelvic fracture on mortality after trauma: An analysis of 63,000 trauma patients. Journal of Bone and Joint Surgery American.

[2] Guthrie HC, Owens RW, Bircher MD (2010). Fractures of the pelvis. Journal of Bone and Joint Surgery British.

[3] Papakostidis C, Kanakaris NK, Kontakis G, Giannoudis PV (2009). Pelvic ring disruptions: Treatment modalities and analysis of outcomes. International Orthopaedics.

[4] Takao M, Nishii T, Sakai T, Yoshikawa H, Sugano N (2014). Iliosacral screw insertion using CT-3D-fluoroscopy matching navigation. Injury.

[5] Gardner MJ, Morshed S, Nork SE, Ricci WM, Chip ML, Routt C (2010). Quantification of the upper and second sacral segment safe zones in normal and dysmorphic sacra. Journal of Orthopaedic Trauma.

[6] Gardner MJ, Parada S, Routt C (2009). Internal rotation and taping of the lower extremities for closed pelvic reduction. Journal of Orthopaedic Trauma.

[7] Ziran BH, Smith WR, Towers J, Morgan SJ (2003). Iliosacral screw fixation of the posterior pelvic ring using local anaesthesia and computerised tomography. Journal of Bone and Joint Surgery British.

[8] Bishop JA, Routt ML (2012). Osseous fixation pathways in pelvic and acetabular fracture surgery: Osteology, radiology, and clinical applications. The Journal of Trauma and Acute Care Surgery.

[9] Carlson DWA, Scheid DK, Maar DC, Baele JR, Kaehr DM (2010). Safe placement of S1 and S2 iliosacral screws: The “vestibule” concept. Journal of Orthopaedic Trauma.

[10] Mendel T, Noser H, Wohlrab D, Stock K, Radetzki F (2011). The lateral sacral triangle-a decision support for secure transverse sacroiliac screw insertion. Injury.

[11] Routt ML, Simonian PT, Agnew SG, Mann FA (1996). Radiographic recognition of the sacral alar slope for optimal placement of iliosacral screws: A cadaveric and clinical study. Journal of Orthopaedic Trauma.

[12] Mehlman CT, Dipasquale TG (1997). Radiation exposure to the orthopaedic surgical team during fluoroscopy: “how far away is far enough?”. Journal of Orthopaedic Trauma.

[13] Singer G (2005). Occupational radiation exposure to the surgeon. The Journal of the American Academy of Orthopaedic Surgeons.

[14] Dimitriou D, Tsai T-Y, Yue B, Rubash HE, Kwon YM, Li G (2016). Side-to-side variation in normal femoral morphology: 3D CT analysis of 122 femurs. Orthopaedics and Traumatology, Surgery and Research.

[15] Bodzay T, Flóris I, Váradi K (2011). Comparison of stability in the operative treatment of pelvic injuries in a finite element model. Archives of Orthopaedic and Trauma Surgery.

[16] Liu GR, Nguyen-Thoi T, Nguyen-Xuan H, Lam KY (2009). A node-based smoothed finite element method (NS-FEM) for upper bound solutions to solid mechanics problems. Computers and Structures.

[17] McLaren DA, Busel GA, Parikh HR, Only A, Patterson J, Gaston BT, McLemore R, Cunningham B (2021). Corridor-diameter-dependent angular tolerance for safe transiliosacral screw placement: An anatomic study of 433 pelves. European Journal of Orthopaedic Surgery & Traumatology.

[18] Hu P, Wu T, Wang HZ, Qi XZ, Yao J, Cheng XD, Chen W, Zhang YZ (2019). Biomechanical comparison of three internal fixation techniques for stabilizing posterior pelvic ring disruption: A 3D finite element analysis. Orthopaedic Surgery.

[19] Schmidt H, Alber T, Wehner T, Blakytny R, Wilke HJ (2009). Discretization error when using finite element models: Analysis and evaluation of an underestimated problem. Journal of Biomechanics.

[20] Ebraheim NA, Xu R, Biyani A, Nadaud MC (1997). Morphologic considerations of the first sacral pedicle for iliosacral screw placement. Spine.

[21] Noojin FK, Malkani AL, Haikal L, Lundquist C, Voor MJ (2000). Cross-sectional geometry of the sacral ala for safe insertion of iliosacral lag screws: A computed tomography model. Journal of Orthopaedic Trauma.

[22] Mendel T, Radetzki F, Wohlrab D, Stock K, Hofmann GO, Noser H (2013). CT-based 3-D visualisation of secure bone corridors and optimal trajectories for sacroiliac screws. Injury.

[23] Cruz HA, Angelis GPD (2013). Sacroiliac secure corridor: Analysis for safe insertion of iliosacral screws. Revista Brasileira de Ortopedia.

[24] Sagi HC, Ordway NR, DiPasquale T (2004). Biomechanical analysis of fixation for vertically unstable sacroiliac dislocations with iliosacral screws and symphyseal plating. Journal of Orthopaedic Trauma.

[25] Salášek M, Jansová M, Křen J, Pavelka T, Weisová D (2015). Biomechanical comparison of a transiliac internal fixator and two iliosacral screws in transforaminal sacral fractures: A finite element analysis. Acta of Bioengineering and Biomechanics.

[26] Fradet L, Bianco RJ, Tatsumi R, Coleman J, Aubin CÉ (2020). Biomechanical comparison of sacral and transarticular sacroiliac screw fixation. Spine Deformity.

[27] Eichenseer PH, Sybert DR, Cotton JR (2011). A finite element analysis of sacroiliac joint ligaments in response to different loading conditions. Spine (Phila Pa 1976).

[28] Lee CH, Hsu CC, Huang PY (2017). Biomechanical study of different fixation techniques for the treatment of sacroiliac joint injuries using finite element analyses and biomechanical tests. Computers in Biology and Medicine.

[29] Ghosh R, Gupta S, Dickinson A, Browne M (2013). Experimental validation of numerically predicted strain and micromotion in intact and implanted composite hemi-pelvises. Proceedings of the Institution of Mechanical Engineers.

[30] Wong LC, Chiu WK, Russ M, Liew S (2013). Experimental testing of vibration analysis methods to monitor recovery of stiffness of a fixated synthetic pelvis: A preliminary study. Key Engineering Materials.

[31] Burval DJ, McLain RF, Milks R, Inceoglu S (2007). Primary pedicle screw augmentation in osteoporotic lumbar vertebrae—biomechanical analysis of pedicle fixation strength. Spine.

[32] Gao MX, Lei W, Wu ZX, Liu D, Shi L (2011). Biomechanical evaluation of fixation strength of conventional and expansive pedicle screws with or without calcium based cement augmentation. Clinical Biomechanics.

